# Spatio-temporal patterns of stomoxyine flies (Diptera: Muscidae) in a forested area of Thailand[Fn FN1]

**DOI:** 10.1051/parasite/2024062

**Published:** 2024-10-04

**Authors:** Watcharadol Yeohsakul, Tanasak Changbunjong, Suppada Kananub, Saree Nakbun, Jumnongjit Phasuk, Supaphen Sripiboon

**Affiliations:** 1 Department of Large Animals and Wildlife Clinical Sciences, Faculty of Veterinary Medicine, Kasetsart University Kamphaeng Saen Nakhon Pathom 73140 Thailand; 2 Department of Pre-Clinic and Applied Animal Science, Faculty of Veterinary Science, Mahidol University Nakhon Pathom 73170 Thailand; 3 Department of Veterinary Public Health, Faculty of Veterinary Medicine, Kasetsart University Kamphaeng Saen Nakhon Pathom 73140 Thailand; 4 Khao Nampu Nature and Wildlife Education Center, Department of National Parks, Wildlife and Plant Conservation Kanchanaburi 71250 Thailand; 5 Department of Parasitology, Faculty of Veterinary Medicine, Kasetsart University Bangkok 10900 Thailand

**Keywords:** Forest, Spatio-temporal, Species diversity, Stomoxyine flies, Thailand

## Abstract

Understanding the distribution patterns of vector populations is crucial for comprehending the dynamics of vector-borne diseases. However, data on vector composition and abundance in areas of forest and wildlife-human interface in Thailand remain limited. This research aimed to investigate the spatio-temporal distribution and species diversity of stomoxyine flies (Diptera: Muscidae) in Salakpra Wildlife Sanctuary, Thailand’s first wildlife sanctuary. A longitudinal entomological survey was conducted monthly from May 2022 to April 2023 in four habitats: core forest, grassland forest, a wildlife breeding center, and a local cattle farm. A total of 11,256 stomoxyine flies from four genera were captured. Based on morphological keys, nine species of stomoxyine flies were identified: *Stomoxys pullus* (29.63%), *Stomoxys calcitrans* (19.65%), *Stomoxys indicus* (16.09%), *Haematostoma austeni* (14.23%), *Haematobia irritans exigua* (8.22%), *Haematobosca sanguinolenta* (7.96%), *Stomoxys uruma* (1.98%), *Stomoxys sitiens* (1.75%), and *Stomoxys bengalensis* (0.49%). Heterogeneous variations in abundance across months and habitats were observed, in which abundance increased in the rainy season (June–October), exhibiting bimodal peaks at seasonal transitions. Human-disturbed areas, such as the cattle farm, exhibited the highest density and species diversity of stomoxyine flies. In contrast, areas with minimal human disturbance, like core forest, had low diversity and density but supported unique species, like the abundant *Haematostoma austeni,* which had minor populations in other types of habitats. The results of this study can be integrated into epidemiological models and lay the groundwork for more comprehensive research on vector-borne diseases at the wildlife-livestock interface to mitigate transmission risks and preserve biodiversity.

## Abstract (fr)

Comprendre les schémas de distribution des populations de vecteurs est essentiel pour comprendre la dynamique des maladies à transmission vectorielle. Cependant, les données sur la composition et l’abondance des vecteurs dans les zones de forêt et d’interface faune-humain en Thaïlande restent limitées. Cette recherche visait à étudier la distribution spatio-temporelle et la diversité des espèces de stomoxes (Diptera : Muscidae) dans le sanctuaire faunique de Salakpra, le premier sanctuaire faunique de Thaïlande. Une enquête entomologique longitudinale a été menée mensuellement de mai 2022 à avril 2023 dans quatre habitats : forêt centrale, forêt de prairie, un centre d’élevage d’animaux sauvages et une ferme d’élevage locale. Au total, 11 256 stomoxes de quatre genres ont été capturés. Sur la base des clés morphologiques, neuf espèces de stomoxes ont été identifiées : *Stomoxys pullus* (29,63 %), *Stomoxys calcitrans* (19,65 %), *Stomoxys indicus* (16,09 %), *Haematostoma austeni* (14,23 %), *Haematobia irritans exigua* (8,22 %), *Haematobosca sanguinolenta* (7,96 %), *Stomoxys uruma* (1,98 %), *Stomoxys sitiens* (1,75 %) et *Stomoxys bengalensis* (0,49 %). Des variations hétérogènes de l’abondance selon les mois et les habitats ont été observées, l’abondance augmentant pendant la saison des pluies (juin à octobre), présentant des pics bimodaux aux transitions saisonnières. Les zones perturbées par l’homme, comme la ferme d’élevage, présentaient la plus forte densité et la plus grande diversité d’espèces de stomoxes. En revanche, les zones peu perturbées par l’homme, comme la forêt centrale, présentaient une faible diversité et une faible densité, mais abritaient des espèces uniques, comme l’abondant *Haematostoma austeni*, dont les populations étaient mineures dans d’autres types d’habitats. Les résultats de cette étude peuvent être intégrés dans des modèles épidémiologiques et posent les bases d’une recherche plus complète sur les maladies à transmission vectorielle à l’interface faune-bétail afin d’atténuer les risques de transmission et de préserver la biodiversité.

## Introduction

Stomoxyine flies (Diptera: Muscidae) have a significant impact on both medical and veterinary science. This group comprises 51 species within 10 genera, with five genera (*Stomoxys*, *Haematobosca*, *Haematobia*, *Haematostoma*, and *Stygeromyia*) being particularly notable pests in livestock [25]. These flies primarily target large mammals and are known to transmit a wide range of pathogens [48]. Besides serving as nuisance pests due to their painful bites and blood-feeding behavior, they act as mechanical vectors for various pathogens, including viruses (e.g., bovine leukosis virus, lumpy skin disease virus, and West Nile virus), rickettsia, bacteria, and parasites, including *Trypanosoma evansi*, the agent of surra, a disease largely distributed notably in Thailand [3, 14, 17, 42, 43]. Additionally, some species serve as biological vectors for organisms such as filarial nematodes, *Trypanosoma* spp., and apicomplexans [3, 44].

The emergence of infectious zoonotic diseases, especially those transmitted by vectors, is aggravated by climate change and the encroachment of agricultural land into forest-protected areas. These environmental changes directly impact the abundance and distribution of vectors, thereby influencing disease transmission dynamics [5, 40]. Recent outbreaks of vector-borne diseases, such as lumpy skin disease (LSD) in Thailand, which affects both domestic and wild populations, underscore the complex interface between humans, wildlife, and livestock [2, 38]. Illegal cattle grazing in protected forest areas not only competes with wild herbivores for resources, but also introduces infectious diseases into wildlife populations, creating a nexus for disease transmission between domestic and wild animals [4]. Despite the potential for disease transmission in this interface area, research on arthropod vectors, particularly blood-sucking flies, within natural environments remains limited.

Previous research on stomoxyine flies in Thailand has focused predominantly on spatial dynamics, while temporal aspects have largely been neglected. Furthermore, previous studies have been confined mainly to agricultural settings [9, 25–27, 32, 37]. Earlier studies showed that the stable fly (*Stomoxys calcitrans*) was the most predominant species and had a wide distribution in Thailand, with populations typically peaking during the rainy season and decreasing during the cool season [9, 26, 32, 37]. Forested protected areas have received little attention regarding vector diversity and abundance. A previous study showed that *Stomoxys pullus* has emerged as the dominant species within one of the forested protected area of Thailand [10], suggesting that different habitats may significantly influence species composition.

The primary objective of this research is to investigate the population dynamics, distribution, seasonal abundance, and species diversity of stomoxyine flies in four distinct areas within or near the Salakpra Wildlife Sanctuary in Thailand. The study seeks to assess how human-altered landscapes and seasonal variations influence the abundance of these flies. The findings will be valuable in advancing research on vector-borne diseases, providing essential data for developing surveillance plans and control strategies.

## Materials and methods

### Ethical statement

The research protocols used for specimen collection in this study received prior approval from the Kasetsart University Institutional Animal Care and Use Committee (Ethics Approval Number: ACKU65-VET-028). Furthermore, this study was conducted in full compliance with the regulations of the Department of National Parks, Wildlife, and Plant Conservation (DNP 0907.4/15458), Thailand.

### Sampling sites

**Figure 1:**
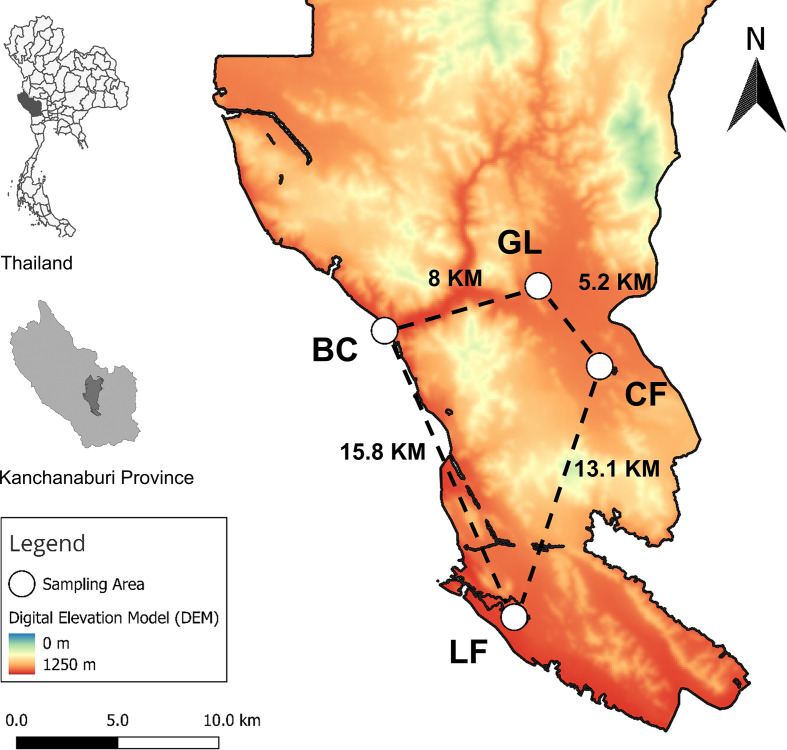
Map of the geographical location and distance between the four sampling sites in Kanchanaburi province, Thailand (CF: core forest, GL: grassland forest, BC: banteng breeding center, LF: local cattle farm).

The selection of Salakpra Wildlife Sanctuary (SWS) was driven by its importance in wildlife conservation, particularly banteng (*Bos javanicus*), and its interface between domestic animals and wildlife, increasing the risk of disease outbreak in this area [6]. Based on habitat types, host distribution, and human disturbance gradient, four sampling sites were selected within or near the SWS (Fig. 1). The details of each sampling site are described below.

**Figure 2:**
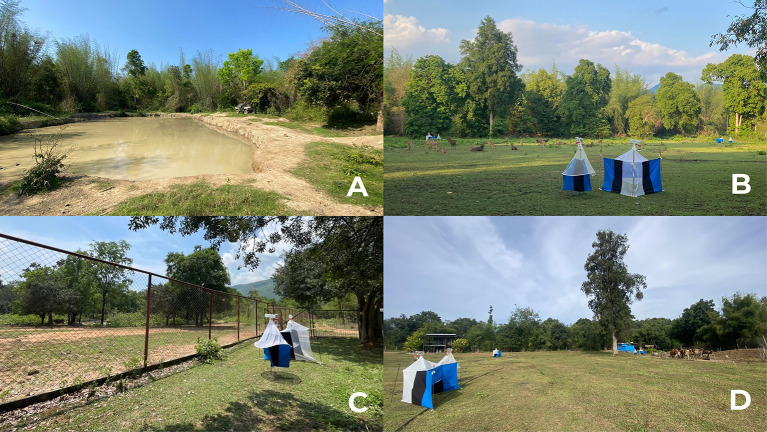
Landscape of each sampling site (A: core forest (CF), B: grassland forest (GL), C: banteng breeding center (BC), D: local cattle farm (LF)) and the side by side trap setting.

- Core Forest (CF) (14°18′31.2″N, 99°18′13.3″E; Fig. 2A): located in the SWS, dominated by a mixed deciduous forest with a closed canopy. It contains an artificial pond and natural mineral lick that attract a diverse range of wildlife, including herds of Asian elephants (*Elephas maximus*), a herd of reintroduced banteng, sambar deer (*Rusa unicolor*), and barking deer (*Cervus muntjak*). This area remains minimally disturbed by human activities.
- Grassland forest (GL) (14°20′42.3″N, 99°16′37.6″E; Fig. 2B), located in the central part of the SWS, characterized by expanses of open terrain dominated by grasses. It was used for releasing reintroduced banteng and is currently inhabited by herds of Eld’s deer (*Rucervus eldii*). The presence of the Salakpra Ranger Guard Station in this area results in low levels of human disturbance.
- Banteng Breeding Center (BC) (14°19′27.6″N, 99°12′27.6″E; Fig. 2C), part of the Khao Nampu Nature and Wildlife Education Center (KNP), located on the edge of the SWS. This center houses approximately 16 banteng for captive breeding and reintroduction programs. Adjacent to the main road and villages, primarily engaged in cattle and water buffalo farming, thus the area is set as an intermediate level of human disturbance.
- The local farm (LF) (14°11′48.6″N, 99°15′56.3″E; Fig. 2D) located 15.8 km from the banteng breeding center (BC) and directly adjacent to the edge of the SWS. The farm accommodates 16 cattle within a fenced enclosure. Throughout the sampling period, no animal movements were observed, and no insecticides were used. The village surrounding this local farm is characterized by traditional poultry, cattle, goat, and water buffalo farms, contributing to a high level of human disturbance in this sampling site.

Based on data from the Thai Meteorological Department, the climate in the study area during the research period can be categorized into three seasons: the hot season (February to June), the rainy season (June to October), and the cool season (October to February). In addition, data retrieved from the nearest meteorological station in Kanchanaburi province revealed that the average annual temperature during the study period was 28.3 °C, with a mean annual rainfall of 1431 mm yr^−1^.

### Sampling strategy

Flies were collected monthly over a one-year period, from May 2022 to April 2023, using Nzi traps [30] and Vavoua traps [21]. These traps were locally made and constructed from blue polyester fabric (CR Solon No.41), which has been tested previously for attracting biting flies [34]. Each sampling site (CF, GL, BC, and LF) consisted of three sampling points. At each sampling point, the Nzi and Vavoua traps were placed side by side to maximize the capturing rate of different species [13], and were oriented toward open areas with host presence. Each sampling point was approximately 50 m apart from the other. Therefore, six traps (three Nzi traps and three Vavoua traps) were placed at each sampling site.

Each month, the traps were placed simultaneously in all habitats and remained active for three consecutive days. Temperature (°C) and relative humidity (%RH) were recorded during trap installation at all study sites using iButton^®^ (DS1923, Maxim Integrated Products, Inc., San Jose, CA, USA). The trapped insects were preserved in 95% ethanol, used as a killing agent. The sample containers were labeled and transported at ambient temperature to the Faculty of Veterinary Medicine of Kasetsart University for species identification. Stomoxyine flies were identified at the species level using Zumpt [48] and Tumrasvin and Shinonaga [45] morphological keys.

### Data analysis

The number of flies was calculated as flies per trap per day (FTD) to standardize the data and estimate the abundance using a bellowed formula.

$$\mathrm{FTD}=\frac{\mathrm{Number}\;\mathrm{of}\;\mathrm{captured}\;\mathrm{flies}}{\mathrm{Number}\;\mathrm{of}\;\mathrm{traps}\;\times \;\mathrm{Number}\;\mathrm{of}\;\mathrm{days}\;\mathrm{of}\;\mathrm{capture}}$$

For measuring species diversity, two indices were applied: the Shannon–Wiener index $\left[\mathrm{H'}=-\sum (P_{i}\;\times \;\ln(P_{i}))\right]$ and Simpson’s diversity index $\left[D=1-\sum (P_{i}^{2})\right]$, where *P*_*i*_ is the proportion of species *i* relative to the total number of species [20]. R software [39] was used for statistical analysis. To normalize FTD, a log_10_ transformation: *Y* = log_10_(*X* + 0.1) was performed. Log-transformed data were used for all analyses. Linear models were used to test the influence of space (habitat), time (month), and their interaction on relative abundance for each group. Initially, each variable was tested in a univariate analysis and considered the significant level at 0.2. All identified variables were included in a multivariate model and implemented by backward stepwise regression to select a subset of variables for a final linear regression. The analyses considered the significant level at 0.05. The interaction effects were examined to understand their collective impact. Model selection criteria were based on the Akaike information criterion (AIC), with preference given to the model exhibiting the minimum AIC value [15]. To ensure the validity of the multivariable model, five critical assumptions were assessed, including linearity, multivariate normality, multicollinearity, autocorrelation, and homoscedasticity. A Shapiro–Wilk normality test was used to test the normality of residuals. Variance Inflation Factor (VIF) was conducted to detect and mitigate any problematic multicollinearity among independent variables.

## Results

**Figure 3:**
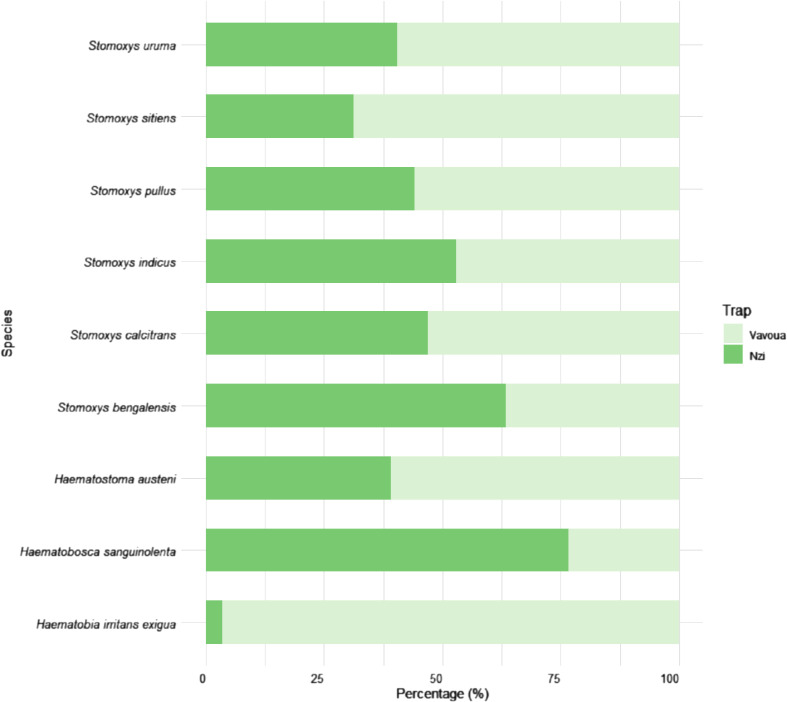
Proportion of stomoxyine flies by trap type, indicating the efficiency of each trap type.

A total of 11,256 stomoxyine flies were captured between May 2022 and April 2023 (795 trap-days). Additionally, due to flooding, samples in the forested area (CF and GL) could not be collected in October, together with some traps that were damaged by wild elephants during the sampling period, resulting in missing data (*n* = 69 trap-days) for certain months. Details of monthly trap loss, temperature, and humidity during the sampling period are presented in Table 1. Species identification revealed nine species of stomoxyine flies from four genera, including *Stomoxys pullus* Austen (29.63%), *Stomoxys calcitrans* L. (19.65%), *Stomoxys indicus* Picard (16.09%), *Haematostoma austeni* Malloch (14.23%), *Haematobia irritans exigua* Bezzi (8.22%), *Haematobosca sanguinolenta* Austen (7.96%), *Stomoxys uruma* Shinonaga & Kano (1.98%), *Stomoxys sitiens* Rondan (1.75%), and *Stomoxys bengalensis* Picard (0.49%) (Table 2). Even though the traps were installed side by side, there was variation in the number of flies captured depending on the trap types (Fig. 3). The Nzi trap showed a high proportion of captures for *H. sanguinolenta* (76.63%), while the Vavoua trap showed a high proportion of captures for *H. irritans exigua* (96.51%) and *S. indicus* (68.65%).

**Table 1:** Details on traps-days, temperature, and relative humidity during trap installation for each season (Hot season = February to June, Rainy season = June to October, and Cool season = October to February) at each type of habitat (CF: Core Forest, GL: Grassland Forest, BC: Banteng Breeding Center, LF: Local Farm).

Habitat	Season	Traps-days		Temperature (°C)			Relative humidity (%)		
		Total	Missing	Max	Mean	Min	Max	Mean	Min
LF	Hot	54	0	50.5	30.8	18.6	99.8	56.2	17.7
	Rainy	90	0	49.1	28.8	23.1	101.3	80.3	28.8
	Cool	72	0	49.5	28.2	18.6	100.8	72.9	22.4
BC	Hot	54	0	50.1	30.2	19.6	98.0	58.5	14.6
	Rainy	90	0	50.5	28.6	23.1	102.8	81.7	27.6
	Cool	72	0	46.1	28.2	18.6	98.5	69.9	22.8
GL	Hot	54	0	51.0	29.3	15.1	99.4	60.1	15.1
	Rainy	69	21	47.6	28.5	22.6	101.7	78.7	27.8
	Cool	63	9	51.0	26.5	16.6	102.8	78.5	18.6
CF	Hot	54	0	45.0	28.6	21.1	98.4	67.7	18.2
	Rainy	60	30	43.6	25.9	22.6	103.3	92.2	35.4
	Cool	63	9	43.1	24.6	15.6	99.8	80.9	30.5

**Table 2:** The average number of flies per trap per day (FTD) of stomoxyine flies for each season (Hot season = February to June, Rainy season = June to October, and Cool season = October to February) at each type of habitat (CF: Core Forest, GL: Grassland Forest, BC: Banteng Breeding Center, LF: Local Farm).

Species	Flies per trap per day ± SD							
	Sampling sites				Season			Total
	CF	GL	BC	LF	Hot	Rainy	Cool	
*Haematobia irritans exigua* (*n* = 958)	0±0.02	0.22±0.29	1.18±1.77	3.05±5.57	0.51±1.12	1.95±4.56	0.75±2.02	1.16±3.18
*Haematobosca sanguinolenta* (*n* = 922)	0.01±0.03	0.22±0.41	0.35±0.45	3.73±9.1	0.07±0.19	2.47±7.55	0.39±1.0	1.12±4.84
*Haematostoma austeni* (*n* = 1572)	1.38±1.65	2.93±4.27	2.45±2.67	1.28±1.35	0.75±0.95	2.97±2.77	1.85±3.25	2±2.75
*Stomoxys bengalensis* (*n* = 57)	0±0	0.11±0.18	4.16±5.9	6.35±9.12	0.05±0.18	0.09±0.18	0.04±0.17	0.06±0.18
*Stomoxys calcitrans* (*n* = 2292)	0±0	0.01±0.04	0.11±0.19	0.14±0.27	1.37±3.18	4.87±8.86	1.45±2.19	2.77±6.11
*Stomoxys indicus* (*n* = 1860)	0±0	0.05±0.15	0.25±0.42	0.64±1.16	1.34±3.02	4.45±7.11	0.51±0.9	2.27±5.02
*Stomoxys pullus* (*n* = 3165)	0.19±0.36	7.32±12.79	5.43±8.93	3.69±5.73	0.69±0.92	6.82±8.13	3.81±11.02	4.18±8.54
*Stomoxys sitiens* (*n* = 201)	0.07±0.14	4.04±7.41	2.17±4.69	2.75±4.45	0.35±1.09	0.26±0.47	0.14±0.42	0.24±0.67
*Stomoxys uruma* (*n* = 229)	0±0.02	0.19±0.22	0.37±0.63	0.5±0.56	0.1±0.19	0.54±0.62	0.11±0.26	1±4.09
Total	0.18±0.7	1.68±5.59	1.83±4.36	2.46±5.54	0.58±1.66	2.71±5.94	0.27±0.48	1.56±4.6

### Spatial distribution

**Figure 4:**
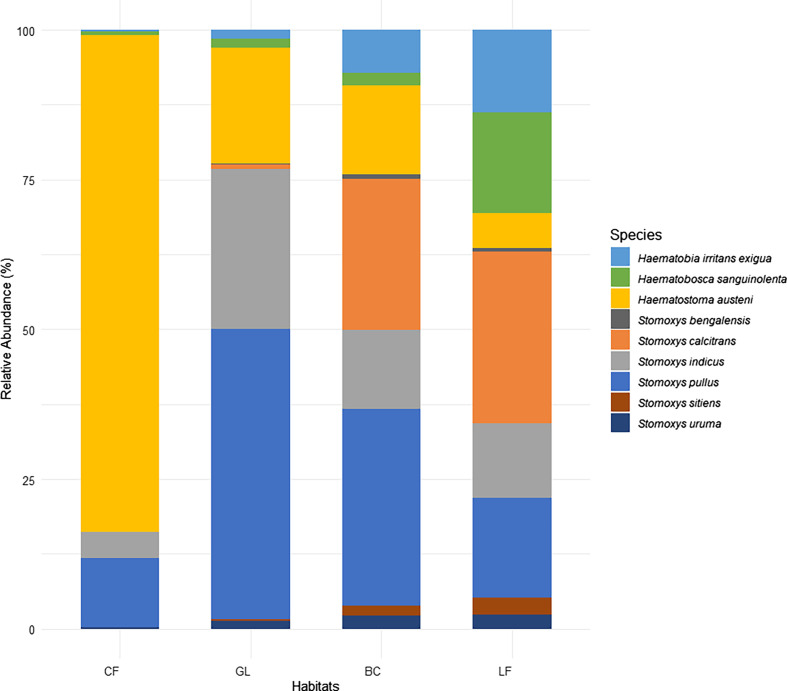
Relative abundance (RA) of each stomoxyine fly at each sampling site (CF: core forest, GL: grassland forest, BC: banteng breeding center, LF: local cattle farm).

The abundance of stomoxyine flies varied between habitats, with the highest collections recorded in LF (41%), followed by BC (30.53%), GL (25.65%), and CF (2.82%). The dominant species also varied by habitat (Fig. 4): *S. calcitrans* was predominant in LF (28.65%) and BC (25.2%), *H. austeni* was dominant in CF (83%), and *S. pullus* was mainly captured in GL (48.4%). A spatial distribution pattern for stomoxyine flies was observed, corresponding to human disturbance gradients and host presence. *Stomoxys* species were more common in areas with significant human disturbance, such as BC and LF, except for *S. pullus*, which was dominant in grassland areas within the forested habitat, where human disturbance was low.

### Temporal distribution

**Figure 5:**
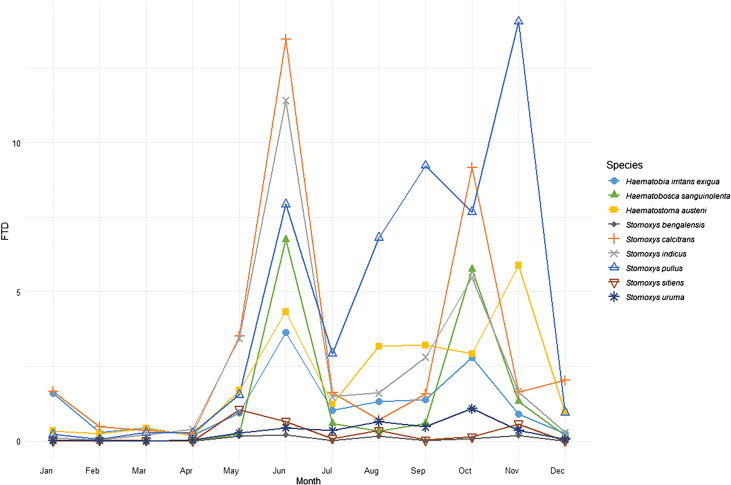
Temporal distribution of stomoxyine flies exhibiting an increasing trend of flies per trap per day (FTD) during the rainy season (June–October) and displaying bimodal peaks at the seasonal transitions.

Most stomoxyine flies were captured during the rainy season (June–October), showing distinct seasonal variation between species (Fig. 5). Stomoxyine flies exhibited two peaks: a major peak during the hot-to-rainy period (June) and a minor peak during the rainy-to-cool period (October), except for *S. pullus* and *H. austeni*, which reached their major peaks at the beginning of cool season (November).

### Species diversity

The diversity indices presented in Table 3, Shannon–Wiener (H’) and Simpson’s (D), offer distinct insights into stomoxyine fly diversity across habitats and seasons. The H’ index measures overall species diversity by accounting for both species richness and evenness, while the D index emphasizes the dominance of particular species. For example, LF exhibited the highest diversity according to both indices (H’ = 1.88; D = 0.82), indicating a well-distributed species composition. Conversely, in CF, although the H’ was low (H’ = 0.59), indicating reduced diversity, the D index highlighted the dominance of *H. austeni* (D = 0.30), reflecting the unique species composition in this area. When considering seasonality, the rainy season demonstrated the highest diversity by both indices (H’ = 1.84; D = 0.81). In contrast, the cool season, despite a relatively high H’ value (H’ = 1.81), exhibited a much lower D value (D = 0.48), signifying the particular dominance of *S. pullus* during this period. These differences between the indices underscore the importance of considering both metrics in ecological assessments, as they reveal complementary aspects of species distribution.

**Table 3:** Species diversity index, Shannon–Wiener and Simpson’s indexes of total stomoxyine flies for each season and habitat.

	Diversity index	
	Shannon-Wiener (H’)	Simpson’s (D)
**Habitat**		
• Core Forest	0.59	0.30
• Grassland Forest	1.28	0.67
• Banteng Breeding Center	1.72	0.78
• Local Farm	1.88	0.82
**Season**		
• Hot (February to June)	1.70	0.77
• Rainy (June to October)	1.84	1.84
• Cool (October to February)	1.81	1.81

### Spatiotemporal distribution

**Figure 6:**
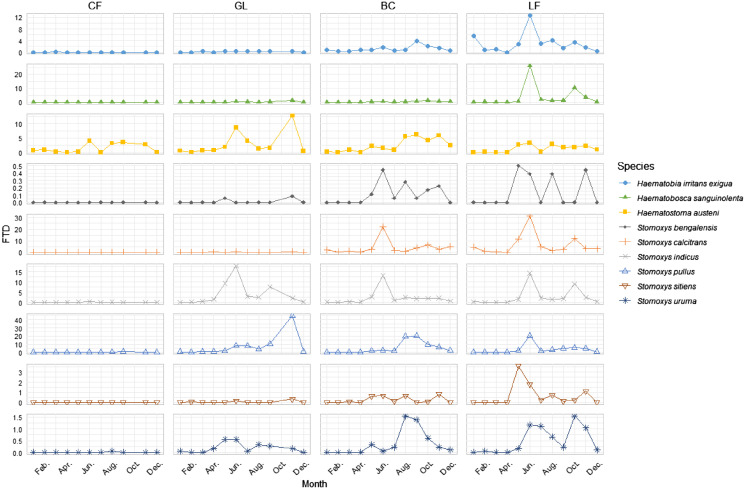
Spatial and temporal variations of stomoxyine flies showing the variation in abundance across habitats, in flies per trap per day (FTD). The LF had peaks at the beginning and end of the rainy season, BC showed unimodal peaks at different times for each species, GL had a bimodal peak mainly in the cool season, and CF primarily had *H. austeni*, increasing during the rainy season without a distinct peak (CF: core forest, GL: grassland forest, BC: banteng breeding center, LF: local cattle farm).

Spatiotemporal variations in population dynamics were evident across each habitat (Fig. 6). Stomoxyine flies exhibited heterogeneous abundance patterns across different habitats and months. At the LF, a consistent pattern emerged, with most fly species showing a major peak at the beginning of the rainy season (May–June) and a minor peak at the end of the rainy season (October–November). In contrast, within BC, each species reached its unimodal peak at different times, indicating a lack of uniformity in the population dynamics. In addition, *S. calcitrans* and *S. indicus* reached their single peak abundance at the beginning of the rainy season (June), while other species peaked later (August–September). In GL, a bimodal peak was observed, with most species, except *S. indicus*, reaching their prominent peak at the beginning of the cool season (November). Interestingly, in CF, where *H. austeni* was the dominant species, no distinct peak was observed; instead, it maintained relatively stable levels throughout the rainy season. When considering individual stomoxyine species, each showed different patterns across habitats; for instance, *S. pullus* and *H. austeni* reached their peaks at different times depending on the habitat. These heterogeneous patterns underscore the complexity of the dynamics of stomoxyine fly populations in various habitats.

### Influence of habitat, month, and their interaction on relative abundance

**Figure 7:**
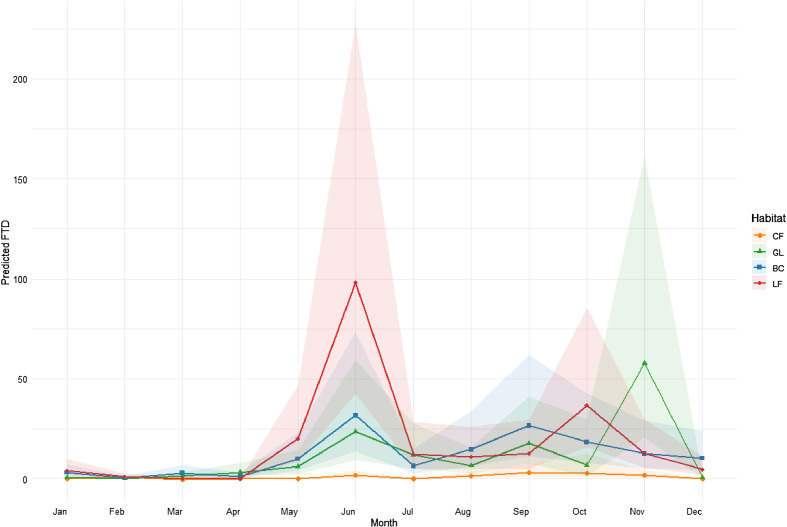
Predicted stomoxyine fly abundance (flies per trap per day: FTD) by habitat across months with confidence intervals. The solid line represents the mean predicted abundances and the shaded area represents the confidence interval (CF: core forest, GL: grassland forest, BC: banteng breeding center, LF: local cattle farm).

Our model selection procedure found that the best model for explaining the variation in stomoxyine fly abundance included habitat, month, and their interactions (adjusted *R*^2^: 0.72, *p* = 0.001) (Fig. 7). This suggested that both temporal and spatial factors significantly influenced fly populations. The results showed that human-disturbed areas, such as BC and LF, had a notably positive effect on fly abundance (*p* = 0.001). Fly populations generally increased from May to November, during the rainy and transition seasons, and decreased from December to April, covering the cool to hot seasons. Specific months with significant positive impacts were June, August, September, October, and November (*p* = 0.001).

Interactions between month and habitat varied. In CF, fly populations remained low throughout the year. GL showed increased abundance from April to November, with significant peaks in June and November (*p* < 0.05). BC had notable peaks in June and September, and higher abundance in February, March, May, and December (*p* < 0.01). LF experienced significant peaks in June (*p* < 0.01) and a minor peak in October, with notable decreases in February and April (*p* < 0.05), and increases in May, June, and July (*p* < 0.05). These findings highlight the complex relationship between habitat and month, which influences fly population dynamics in diverse ways.

## Discussion

This study was the first research to monitor the population dynamics of stomoxyine flies within a forest protected area in Thailand, specifically focusing on the Salakpra Wildlife Sanctuary and its adjacent area. The primary objective was to improve understanding of the distribution, abundance, and seasonality of stomoxyine flies in protected forest areas in Thailand. The investigation revealed the presence of nine species within four genera of stomoxyine flies. Although 11 species of stomoxyine flies had previously been documented in Thailand [35, 45, 48], this study identified only nine species. Notably absent from the findings from this study were two species, *Haematobosca aberrans* and *Haematobia minuta*. Both are rare species with only few reports found in Thailand [7, 45, 48]. However, differences in vector species composition and abundance between this study and previous research could be due to factors such as sampling location, timeframes, seasons, years, and methodologies, including trap types, quantities, and durations. These factors should be considered in comparative analyses [29, 41]. Additionally, the observed abundance may not accurately reflect true relative abundance due to variations in trapping efficiency, influenced by environmental factors and species-trap attractiveness [16, 21, 30]. For example, low abundance in CF could be due to dense vegetation reducing luminosity, which in turn decreases trapping efficiency. Increased luminosity typically enhances trap effectiveness, while dense vegetation may hinder it [12]. Using olfactory attractants like octenol, cow urine, and phenols can improve trap efficiency [18, 31, 46], but this approach was not used in this study to avoid attracting other wildlife that could disrupt traps.

This study employed the side-by-side trap placing method, as described in a previous study [13], to increase the number of insects captured. It is important to note that this trap installation strategy could influence insect abundance, which should be considered before conducting any comparative studies. Previous studies have highlighted the efficacy of the Vavoua trap in capturing *Stomoxys* flies, while the Nzi trap is more suitable for capturing tabanids [22, 46, 47]. In this study, the Vavoua trap predominantly captured *H. irritans exigua* and *S. indicus*. In contrast, species like *H. sanguinolenta*, were predominantly captured by the Nzi trap. However, since the trapping strategies used in this study may differ from those in other studies, further investigation into the efficiency of these traps for specific species is recommended.

Several studies have been conducted on stomoxyine flies in Thailand, primarily focusing on biology, species diversity, and distribution in agricultural settings [9, 25–27, 32, 37]. This study revealed heterogeneous variations in abundance across different seasons and habitats. Distinct dominant species and varied population dynamics were observed, highlighting the unique ecological niches each species occupies. Specifically, in forested areas, *S. pullus* and *H. austeni* were dominant species. Both were classified as wildlife-related, consistent with previous reports from natural areas [9, 10, 45]. In addition, *H. austeni,* which has infrequently been reported, was predominantly found in the core forest areas of this study; while *S. pullus,* previously reported in Khao Yai National Park in Thailand [10], showed high abundance in grassland areas within the forest in this study.

In areas with higher human disturbance, such as LF and BC, stomoxyine flies showed greater species diversity and abundance, capturing approximately 70% of total specimens. *Somoxys calcitrans*, *S. bengalensis*, *S. sitiens*, *S. uruma*, *H*. *sanguinolenta*, and *H. irritans exigua* were more abundant in these disturbed areas, reflecting their dependence on human activities and livestock [48]. These findings indicate that landscape variations lead to diverse distributions of stomoxyine flies [23], and human disturbances impact their abundance differently. Consequently, human encroachment can negatively affect forest species like *S. pullus* and *H. austeni* [29], contributing to the differences in dominant species and species composition across habitats.

This study revealed seasonal variation in the abundance of stomoxyine flies. Most stomoxyine species were primarily abundant during the rainy season, with bimodal peaks occurring in seasonal transitions, either from the hot to the rainy season (May–June) or from the rainy to the cool seasons (October–November). This pattern is similar to what has been reported for many Dipteran species [1]. However, this pattern differed from some previous studies in Thailand where unimodal peaks were typically observed in August or September [19, 24, 32, 36, 37]. The population dynamics of stomoxyine flies may be linked to local environmental factors, both abiotic and biotic. Abiotic factors such as temperature, humidity, rainfall, and light intensity played crucial roles in creating suitable breeding sites and improving survival rates [9, 19, 27]. Biotic factors, including the density and diversity of blood meal hosts, also influence fly diversity [28]. These flies generally prefer to feed on large mammals such as cattle, horses, and deer [48]. Hence, higher densities of cattle and banteng in captive habitats (BC and LF) attract more flies compared to natural habitats (GL and CF), where hosts are more varied and roam freely.

This study revealed significant spatiotemporal variations in the distribution of stomoxyine flies within Salakpra Wildlife Sanctuary and its surroundings in Thailand. These variations can impact pathogen transmission, particularly when vector populations are high. The findings highlight the need to consider habitat and seasonal influences on vector species composition and abundance to better understand pathogen dynamics. While this study provides valuable data, it focuses solely on stomoxyine flies. The roles of other blood-feeding flies, such as *Musca crassirostris*, tabanids, and hippoboscids in disease transmission are not well understood [8, 11, 33] and require further research. Future studies should prioritize blood meal analysis and vector competence to elucidate the roles of these other flies in diseases transmission, which is essential for developing effective management strategies for vector-borne diseases in forest protected areas.
